# Application Prospect of Ion-Imprinted Polymers in Harmless Treatment of Heavy Metal Wastewater

**DOI:** 10.3390/molecules29133160

**Published:** 2024-07-02

**Authors:** Mengzhen Du, Zihao Xu, Yingru Xue, Fei Li, Jingtao Bi, Jie Liu, Shizhao Wang, Xiaofu Guo, Panpan Zhang, Junsheng Yuan

**Affiliations:** 1 Engineering Research Center of Seawater Utilization Technology of Ministry of Education, School of Chemical Engineering, Hebei University of Technology, Tianjin 300401, China; m15935191859@163.com (M.D.); 17832580716@163.com (Z.X.); xyingru17917@163.com (Y.X.); jingtaob@gmail.com (J.B.); liujie@hebut.edu.cn (J.L.); shizhaow@163.com (S.W.); 2009024@hebut.edu.cn (X.G.); zhangpanpan@hebut.edu.cn (P.Z.); jsyuan2012@126.com (J.Y.); 2 Hebei Collaborative Innovation Center of Modern Marine Chemical Technology, Tianjin 300401, China

**Keywords:** heavy metal ions, ion-imprint, adsorption, pollutants

## Abstract

With the rapid development of industry, the discharge of heavy metal-containing wastewater poses a significant threat to aquatic and terrestrial environments as well as human health. This paper provides a brief introduction to the basic principles of ion-imprinted polymer preparation and focuses on the interaction between template ions and functional monomers. We summarized the current research status on typical heavy metal ions, such as Cu(II), Ni(II), Cd(II), Hg(II), Pb(II), and Cr(VI), as well as metalloid metal ions of the As and Sb classes. Furthermore, it discusses recent advances in multi-ion-imprinted polymers. Finally, the paper addresses the challenges faced by ion-imprinted technology and explores its prospects for application.

## 1. Introduction

In recent decades, rapid industrialization, agricultural expansion, and urban development have led to the discharge of wastewater containing various hazardous substances [1,2,3]. Among these substances, heavy metal pollutants pose significant threats to human health, ecological stability, and environmental sustainability [4,5,6]. Heavy metal ion pollutants include metal cations, like copper, lead, mercury, and nickel, as well as metal oxygenate anions, such as chromate and arsenate [7]. Heavy metal ions are primarily released during industrial production processes such as mining and metallurgy, electronics, chemicals, and machinery manufacturing. These ions pose environmental and biological risks in water, the atmosphere, and soil [8,9]. The recycling and treatment of heavy metals in wastewater, transforming them into valuable resources, can enhance resource utilization efficiency and promote sustainable resource management [10,11,12].

Presently, the primary methods for treating heavy metal wastewater include chemical precipitation [13,14], ion exchange [15,16,17], electrolysis [18,19], membrane filtration [20,21], and adsorption [22,23,24,25,26]. However, these conventional treatment methods suffer from low efficiency and selectivity, making the efficient removal of heavy metal ions challenging. Adsorption is an efficient method of treating pollutants that relies heavily on solid adsorbents to capture and remove pollutants. Among these methods, the ion blotting technique stands out for its high selectivity and specificity in the effective removal of specific heavy metal ions. Ion-imprinting technology can rapidly treat heavy metal ions in wastewater, improve the efficiency and quality of wastewater treatment, and also facilitate the recovery of heavy metal resources in wastewater and promote resource recycling. As a result, it effectively reduces the heavy metal content in wastewater, reduces environmental pressure, and offers the advantages of simplicity, low energy consumption, and easy recycling. These properties are in line with the requirements of a circular economy and sustainable development, which makes ion blotting technology the most promising water treatment method [27,28]. The principle of heavy metal ion-imprinting is primarily based on ion-imprinting technology, which has evolved from molecular-imprinting technology [29]. In heavy metal ion wastewater treatment, heavy metal ions serve as templates, forming chelates with functional monomers through electrostatic and ligand interactions. This process enables the adsorption and chelation of specific heavy metal ions [30], leading to a reduction in the heavy metal content in wastewater and achieving harmless treatment. The ion-imprinted adsorption method is a type of adsorption method known for its high selectivity and good recycling performance. It can be synthesized in various ways to take advantage of its current research direction [27,28,29,30,31,32,33,34,35]. Table 1 lists the studies of other materials used for adsorption to remove heavy metal ions. The adsorption capacity of modified carbon materials (e.g., activated carbon, etc.), zeolites, membrane materials, etc., although extensive, usually lacks a highly specific adsorption capacity for specific heavy metal ions, and the ion-imprinting technology can significantly improve the adsorption capacity and efficiency of adsorbents through precise molecular design and imprinting processes to realize the adsorption capacity and efficiency of adsorbents for low concentrations of heavy metal ions.

This paper provides a review of the synthesis principles and preparation methods of ion-imprinted polymer (IIP) adsorbents, with a specific focus on the preparation of IIPs for classical heavy and toxic metals, including Pb(II), Hg(II), Cd(II), Cr(VI), Cu(II), and Ni(II). The practical applications and their effects are also discussed. Finally, the potential application of ion-imprinted polymers in the safe treatment of heavy metal wastewater is discussed.

## 2. Principles of Ion-Imprinting Technology

Molecular imprinting is a terminology that is characteristic for polymers, where a molecularly imprinted polymer (MIP) is a polymer in which molecular recognition sites for a specific target molecule have been created, aiming to obtain robust materials with high selectivity for that particular target molecule [48]. In other words, the physicochemical interactions between the functional moieties of the polymeric matrix and the functional groups of the target molecule (or the target molecule analogue) are memorized during molecular imprinting, are further cemented during structure stabilization, and are subsequently activated by the extraction of the target molecule. The resulting robust polymer with molecular cavities of a specific shape and electronic environment is an MIP with molecular recognition properties for that particular target molecule.

**Figure 1:**
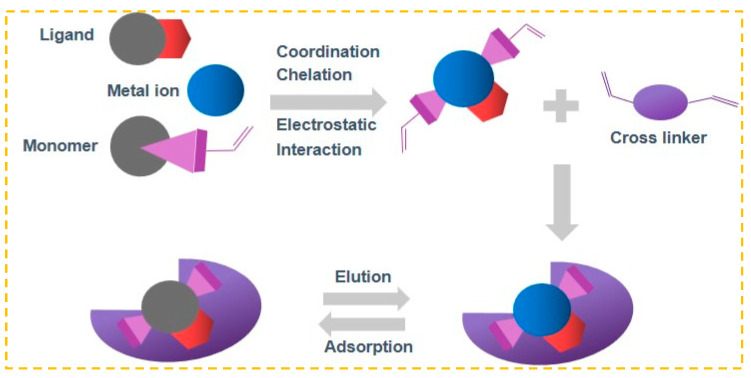
IIP Preparation flow chart.

Ion-imprinting technology, a significant division of molecular-imprinting technology, operates on comparable principles [49]. In Figure 1, the target ion being processed acts as a template and is connected to the ligand through coordination, chelation, or electrostatic interaction. Following the process of cross-linking polymerization, the template ion is removed, resulting in the formation of three-dimensional cavities that contain specific arranged groups and have sizes and shapes that match the target ion [50]. In future practical applications, the desired ion can be specifically identified and captured. Because of its benefits of pre-determined and precise recognition, IIP is also commonly employed for removing heavy metal ions from wastewater or for detecting trace and ultra-trace amounts of ions [51].

Functional monomers are responsible for providing functional groups that can bind to template ions through covalent or non-covalent bonds, as well as end groups that can connect to cross-linking agents [52]. The choice of functional monomers typically relies on the configuration of template ions and the bond formed between functional monomers and template ions. The intensity of this bond and the proportion of functional monomers to template ions in the preparation phase are crucial factors affecting the ions’ affinity and precision [53]. In general, the selected functional monomers should have stable chemical properties and contain unsaturated double bonds and functional groups, such as -COOH, -CHO, -OH, -CONH-, -NH_2_, -SH, etc. Commonly used functional monomers include acrylamide [54], acrylic acid [55], hydroxyethyl methacrylate [56,57], 4-vinylpyridine [58,59], etc.

Certain functional monomers may not be suitable for imprinting or may lack sufficient binding strength, necessitating the use of ligands for assistance. Ligands containing electron-rich heteroatoms, like N, P, S, and O, can engage with unoccupied orbitals in the outer shell of metal ions that have more lone pair electrons. This interaction leads to the formation of chelates, which strengthen the bond between the imprinted polymer (IIP) and template ions. This process enhances the selectivity and precision of adsorption [60]. While ligands are commonly employed in the synthesis of IIPs, there is a significant potential for ligand leakage from the polymer matrix when removing template ions [61]. Common ligands include 2-mercaptobenzothiazole [62], dithizone [63], etc.

In the polymerization process, the cross-linking agent combines with other monomers to create copolymers, which establish a three-dimensional structure and reinforce the binding cavity [64]. The primary purpose of the cross-linking agent in IIP is to stabilize the three-dimensional arrangement of template ions and functional monomers. The type and amount of cross-linking agent used greatly influences the adsorption capabilities of IIP. The cross-linking agents in use are typically categorized into two groups according to their cross-linking mechanisms: One type can interact with functional monomers, like ethylene glycol dimethylacrylate(EGDMA) [54,65,66], N,N′-methylenediamine acrylamide(MBAA) [59,67]; Another category of substances that do not participate in the reaction undergo polymerization by cross-linking in a linear or hyperbranching manner, like glutaraldehyde [14], epichlorohydrin(ECH) [68,69]. In the polymerization process, the cross-linking agent combines with other monomers to create copolymers, which establish a three-dimensional structure and reinforce the binding cavity [64]. Inadequate amounts of cross-linking agent can result in reduced mechanical properties of IIP, an unstable structure of recognition sites, and diminished selectivity. Conversely, an excessive amount can lead to a decline in mass transfer performance and effective recognition sites of IIP, impacting its adsorption capacity and rate [28]. Experimental and computer simulation methods can be used to determine the appropriate cross-linking agents and their ratio to functional monomers for optimization.

Of course, the initiator is also essential in the ion imprinting process. It can not only promote the smooth progress of the polymerization reaction, but also optimize the performance of the imprinted polymer by regulating the polymerization process, so as to ensure that the ion-imprinting technology has the best effect in the treatment of heavy metal wastewater and other applications [64]. Initiators are substances that can initiate a polymerization reaction under certain conditions [70]. Peroxides and azo compounds are commonly used initiators [52]. The proportion of initiators in the polymerization composition is very small, usually determined by the type of polymerization. Since propriety polymerization is characterized by the absence of solvent, the oil-soluble initiator can be dissolved in the monomer and initiate the polymerization. In suspension polymerization, the oil-soluble initiators can be dissolved in organic solvents or oil phases to initiate polymerization and form polymers because the reaction medium is a coexisting system of aqueous and oily phases. benzoyl peroxide (BPO) [55] and azoisobu-tyronitrile (AIBN) [56,57] are commonly used oil-soluble initiators. Water-soluble initiators like persulfate are appropriate for polymerizing lotions and aqueous solutions [71,72]. Furthermore, there are processes such as photoinitiated polymerization, electrically initiated polymerization, and others.

In ion-imprinting techniques, it is critical to select the appropriate solvent to ensure the morphology of the ion-imprinted polymer and the formation of the internal three-dimensional cavity structure. The choice of solvent directly affects the solubility of functional monomers and template ions, which in turn affects the adsorption properties of ion-imprinted polymers. Commonly used solvents include N,N-dimethylformamide (DMF), methanol, acetonitrile, dimethyl sulfoxide, and toluene. These solvents not only help the interaction between functional monomers and template ions but also promote the formation of cavity structures inside the ion-imprinted polymers, thus realizing the efficient adsorption of target heavy metal ions. At the same time, in order to ensure that the ion-imprinted polymers achieve the best adsorption performance during the adsorption process, the solvents used in adsorption should be consistent with those used in polymerization. This can avoid the possible structural changes of the ion-imprinted polymer during solvent replacement, thus maintaining its good adsorption performance.

## 3. Typical Heavy Metal Ion-Imprinted Polymer

### 3.1. Cu(II)-Imprinted Polymers

Cu(II) ions are among the common heavy metal pollutants found in industrial wastewater. Excessive Cu(II) ions not only contribute significantly to environmental pollution and impair water self-purification abilities [73,74], but also pose serious health risks to humans, including harmful effects on the liver and kidneys, increased blood pressure, and respiratory rate [75,76].

**Figure 2:**
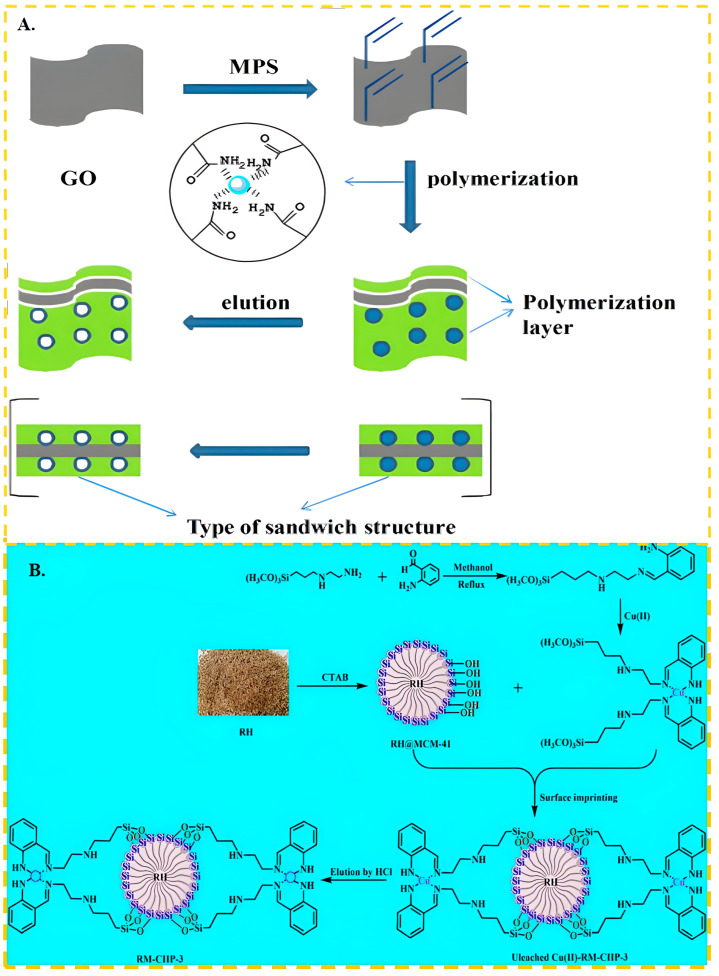
(**A**) Scheme for the synthesis of GO-IIP, adapted with permission from Ref. [54] Copyright 2017 Elsevier; (**B**) Scheme for the synthesis of RM-CIIP-3, adapted with permission from Ref. [95]. Copyright 2022 Elsevier.

At present, in the preparation process of Cu(II) ion-imprinted adsorbent, specific sites are formed by the elution of the amino group and Cu(II) after bonding. The Cu(II) ion can interact with the electron clouds of functional monomers to create a cavity with specific groups. Therefore, functional monomers containing amine groups are commonly employed in the synthesis of Cu(II)-imprinted adsorbents (as illustrated in Table 2). Kong et al. [54] used functional graphene oxide as the carrier, which was modified by 3-(Trimethoxysilyl) propyl methacrylate, acrylamide as the functional monomer, as shown in Figure 2A, and -CONH_2_ was complexed with Cu(II); the maximum adsorption capacity of GO-IIP was up to 132.77 mg/g. Li et al. [77] used N-isopropylacrylamide as the functional monomer, acrylamide as the cross-linking agent, -CONH_2_ and Cu(II) complexation, and tetramethylethylenediamine catalyzed the generation of free radicals from ammonium persulfate to accelerate the polymerization of acrylamide, and finally synthesized the Cu(II)-imprinted polymers (Cu(II)-IIPs) with the maximal adsorption capacity of up to 35.55 mg/g. Fatty amine functional monomers typically exhibit a stronger affinity for binding with Cu(II) ions. This is due to the ability of lone pair electrons in the nitrogen atom of aliphatic amines to form coordination bonds with Cu(II), enabling effective recognition and binding. In contrast, the binding capacity of functional monomers in amine polymers may be weaker, as they often necessitate chelation with Cu(II) through multiple amine groups., and this chelation may be affected by factors such as steric hindrance and flexibility of the polymer chain. About the effect of chain length of fatty amine functional monomer on the adsorption effect of material, Othman et al. [78] used ethylenediamine, diethylene triamine, triethylene tetramine, and tetraethylene pentamine as the functional monomer complexed with complexed, also cross-linked by γ radiation. The results show that short-chain aliphatic amines are more selective, because the complex structural coordination of longer fat chains inhibits the diffusion of Cu(II) in the template cavity.

**Table 2:** Composition and properties of Cu ion-imprinted polymers.

Carrier	Ligand	Functional Monomers	Group	Regeneration Frequencies	Maximum Adsorption Capacity (mg/g)	Ref.
modified palygorskite	NA	NIAM	-CONH- -COOH	6	35.55	[77]
carbon encapsulated Fe_3_O_4_ nanospheres	NA	NIAM	-CONH- -COOH	5	45.46	[72]
Fe_3_O_4_-graphene@mesoporous SiO_2_	NA	TPEMP	R-N	6	195.3	[79]
Montmorillonite	NA	4-VP; MA	C≡N -COOH	8	23.6	[80]
carboxylation CoFe_2_O_4_	NA	POPD	R-N	5	114.198	[81]
poly (glycidyl methacrylate-co-polyethylene glycol dimethacrylate)	DPC	MA	R-N	5	85.6	[82]
NA	NA	Cuphen(VBA)_2_H_2_O	C≡N -COOH	NA	287.45	[83]
epoxy resin	NA	PEPA	-OH -NH	5	91.58	[84]
NA	NA	aloe vera extract	-COOH -OH	8	338.73	[85]
NA	CA	PEI; CMP	-NH_2_	6	87.69	[86]
NA	EDTA	4-VP; MA	C≡N -COOH		2.163	[87]
NA	NA	hydrazine hydrate	C-N C=O	5	312.5	[88]
NA	NA	4-VP; MA	C≡N -COOH	10	26.9	[89]
NA	NA	SA; CTS	-NH_2_	3	83.33	[90]
NA	NA	PEI; HEA	-NH_2_ -NH	5	40	[91]
NA	NA	G; HQ	-OH R-N	10	111.81	[92]
NA	NA	isatin; CTS	R-N -CONH-	5	143	[69]
NA	NA	AAPTMS	-NH_2_ -NH	5	39.82	[93]
nanofiber nonwoven fabric	NA	BC	-OH	10	152.2	[94]

Abbreviations: AM—acrylamide; MA—methacrylic acid; 4-VP—4-vinylpyridine; POPD—polyo-phenylenediamine; OCASBG—Schiff base; PEI—polyethylenimine; DPC—diphenylsemicarbazide; CTS—chitosan; BC—bacterial cellulose; PEPA—polyethylene polyamine; CA—citric acid; PEI—polyethyleneimine; CMP—chloromethylated polystyrene; NIAM—N-isopropylacrylamide; EDTA—ethylenediaminetetraacetic acid disodium salt dihydrate; SA—sodium alginate; HEA—2-hydroxyethyl acrylate; HQ—8-hydroxyquinoline; G—gelatin.

Recently, there has been a growing interest in utilization of ammonium salts as functional monomers. Additionally, researchers have been exploring the modification of ligands to enhance the adsorption capacity and selectivity of Schiff bases, which contain numerous imines or methylimines. This approach also offers insights for the further advancement of Cu(II) ion-imprinting techniques. Ren et al. [93] used N-[3-(2-aminoethylamino) propyl] tri-methoxy-silane as the functional monomer and tetraethyl orthosilicate as the cross-linker; the maximum adsorption capacity of ion-imprinted polymer was 39.82 mg/g and could be reused several times without significant loss of adsorption capacity. As shown in Figure 2B, Gao et al. [95] synthesized the Schiff base OCASBG by mixing [3-(2-Aminoethyl)aminopropyl]trimethoxysilane with 2-aminobenzaldehyde. This functional monomer not only contained imide groups that exhibited improved complexation with Cu(II), but also cross-linked through a dehydration condensation reaction on the surface of the RH@MCM-41 carrier. As a result, the maximum adsorption capacity of RM-CIIP-3 for Cu(II) increased to 91.4 mg/g without the need for additional cross-linking agents.

From Table 2, we can understand that in recent years, Cu(II) ion-imprinted materials based on carboxyl and amine groups have attracted much attention because they are functional groups capable of interacting well with Cu(II) ions, and short-chained aliphatic amines form stable complexes or ligands with Cu(II) ions through their specific chemical properties and structures, which improves the adsorption selectivity of Cu(II) ions. In addition, to improve their adsorption properties, mechanical properties, and recovery from wastewater, different inorganic particles (Fe_3_O_4_, SiO_2_, GO, etc.), have been used to fabricate Cu(II)-IIP-based composites. The Cu(II) ion-imprinted polymer systems have diverse potentials, and researchers have explored the use of a variety of functional monomers, cross-linkers, and additives to enhance the imprinting effects. The aim is to develop a novel Cu(II) ion-imprinted system that addresses the challenges of limited recognition and low adsorption capacity.

### 3.2. Ni(II)-Imprinted Polymers

Ni(II) is a prevalent toxic heavy metal that can enter the human body through skin contact or inhalation, leading to tissue damage and potential risks of nerve poisoning, kidney toxicity, pulmonary fibrosis, heart damage, and cancer [90,96]. The retrieval of Ni(II) is crucial for diminishing the overall Ni(II) levels, thus enhancing environmental safety.

**Figure 3:**
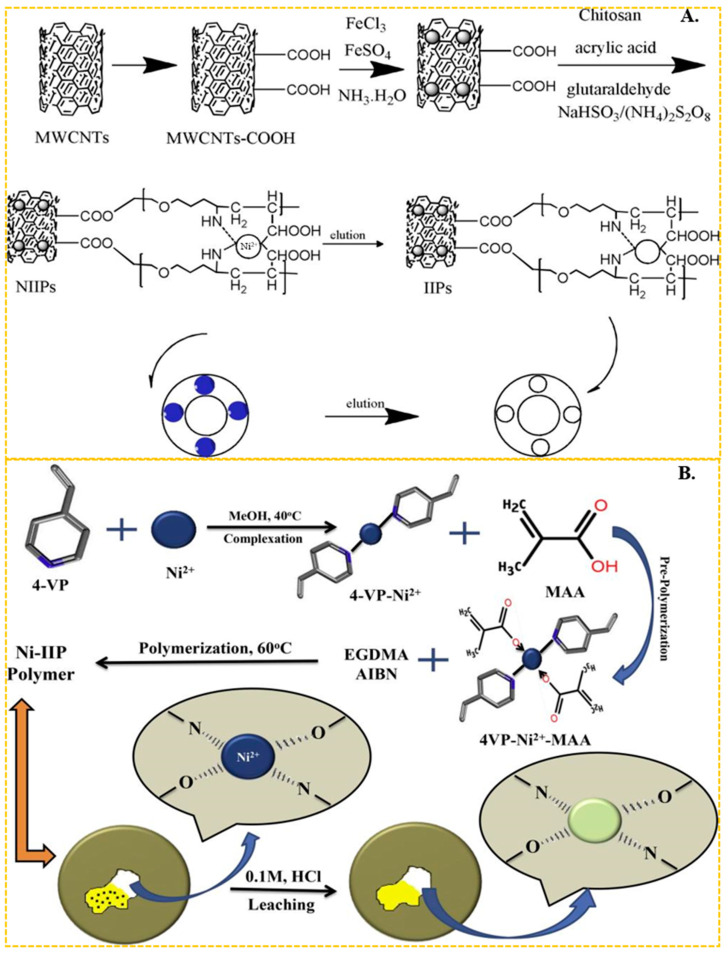
(**A**) Scheme for the synthesis of IIPs, adapted with permission from Ref. [67]. Copyright 2018 Elsevier. (**B**) Scheme for the synthesis of Ni-IIP, adapted with permission from Ref. [55]. Copyright 2019 Elsevier.

In recent years, chitosan has become the most common functional monomer in the preparation of Ni(II) ion-imprinted adsorbent materials. Chitosan features abundant hydroxyl and amino groups on its molecular chain, making it particularly effective at chelating the transition metal nickel. Liu et al. [97] utilized chitosan as the functional monomer and epichlorohydrin as the cross-linker to synthesize a nickel ion-imprinted adsorbent with an adsorption capacity approaching 20.0 mg/g. He et al. [67] used magnetic multi-walled carbon nanotubes as the carrier, N, N-methylbisacrylamide as the cross-linker, crylic acid and chitosan as the functional monomer, through carboxy and amino complexed with Ni(II) and obtained carbon nanotube-based imprinted polymers (IIPs). The preparation process is shown in Figure 3A, the maximum adsorption capacity of the obtained ion-imprinted sorbent was up to 19.86 mg/g. In the above studies using chitosan as functional monomer, the final adsorption capacity of IIPs was about 20 mg/g, which was generally low. It is evident that chitosan, as a cost-effective and readily available green material, still holds significant application.

In addition to the complexation of amino and hydroxyl groups with Ni(II) in chitosan, imine ligands are also common in Ni(II)-imprinted adsorption materials.

Emel Tamahkar et al. [98] used N-methacryloyl-histidine methyl ester as the functional monomer. They complexed it with Ni(II) ions through its imines and synthesized ion-imprinted poly(hydroxyethyl methacrylate)-based supermacroporous cryogels. The maximum adsorption capacity was up to 5.54 mg/g. As shown in Figure 3B, Ameet Kumar et al. [55] complexed Ni(II) ions with the imines of the ligand 4-vinyl and the carboxylic groups of the methacrylic acid (MA) functional monomer. They used ethylene dimethacrylate as a cross-linker. The maximum adsorption capacity of the resulting ion-imprinted sorbent was up to 125 mg/g. Sagar Kumar et al. [66] also employed 4-vinylpyridine as a ligand, MA as the functional monomer in conjunction with Ni(II), and amino-functionalized Fe_3_O_4_@SiO_2_ as the carrier. The maximum adsorption capacity of the synthesized ion-imprinted polymers (IIPs) in a Ni(II) solution with an initial concentration of 20 mg/L can reach 158.73 mg/g. The adsorption capacity of IIPs prepared using a single imide functional monomer is slightly lower. However, good results can be achieved by coordinating imide with carboxyl or oxime groups with Ni(II). In addition, He et al. [99,100] used a 2-acrylamido-2-methyl-1-propanesulfonic acid containing sulfonic acid group as the functional monomer, and complexation with Ni(II) by the sulfonic acid group. This functional monomer was complexed with Ni(II) ions through the sulfonic acid group. The authors employed SG-N-(2-aminoethyl)-3-aminopropyl-trimethoxysilane as a carrier and prepared two types of ion-imprinted polymers (IIPs) using two different cross-linkers, namely ethylene dimeth acrylate and N,N-methylbisacrylamide. The maximum adsorption capacity for Ni(II) ions in a solution with an initial concentration of 100 mg/L was found to be 66.22 mg/g and 20.3 mg/g, respectively, for the two types of IIPs. Remarkably, the adsorption capacity did not exhibit a significant decrease even after six cycles of repeated use.

From Table 3, during the preparation of Ni(II) ion-imprinted adsorption materials, Ni(II) is typically complexed with N atoms in amino and imide groups, as well as O atoms in carboxyl and hydroxyl groups in the functional monomers. The adsorption material obtained by combining imide groups with carboxyl and hydroxyl groups demonstrates superior performance, with an adsorption capacity exceeding 100 mg/g. Moreover, chitosan, a green material, holds great potential in the field of ion-imprinting adsorption, offering ample opportunities for further development.

### 3.3. Cd(II)-Imprinted Polymers

Cd(II) is a highly toxic and non-biodegradable heavy metal. Upon ingestion through the food chain, Cd(II) accumulates in various organs, particularly the kidneys, liver, lungs, bones, and blood, resulting in adverse health effects [113]. Unlike essential elements such as copper and zinc, cadmium poses a threat to human health even at very low concentrations [114]. The International Agency for Research on Cancer (IARC) has classified cadmium as a level 1 carcinogen, while the World Health Organization (WHO) recommends a maximum concentration of 3 μg/L for Cd(II) in drinking water [115,116]. Consequently, the detection of trace amounts of cadmium in water bodies is crucial for effective water pollution management.

**Figure 4:**
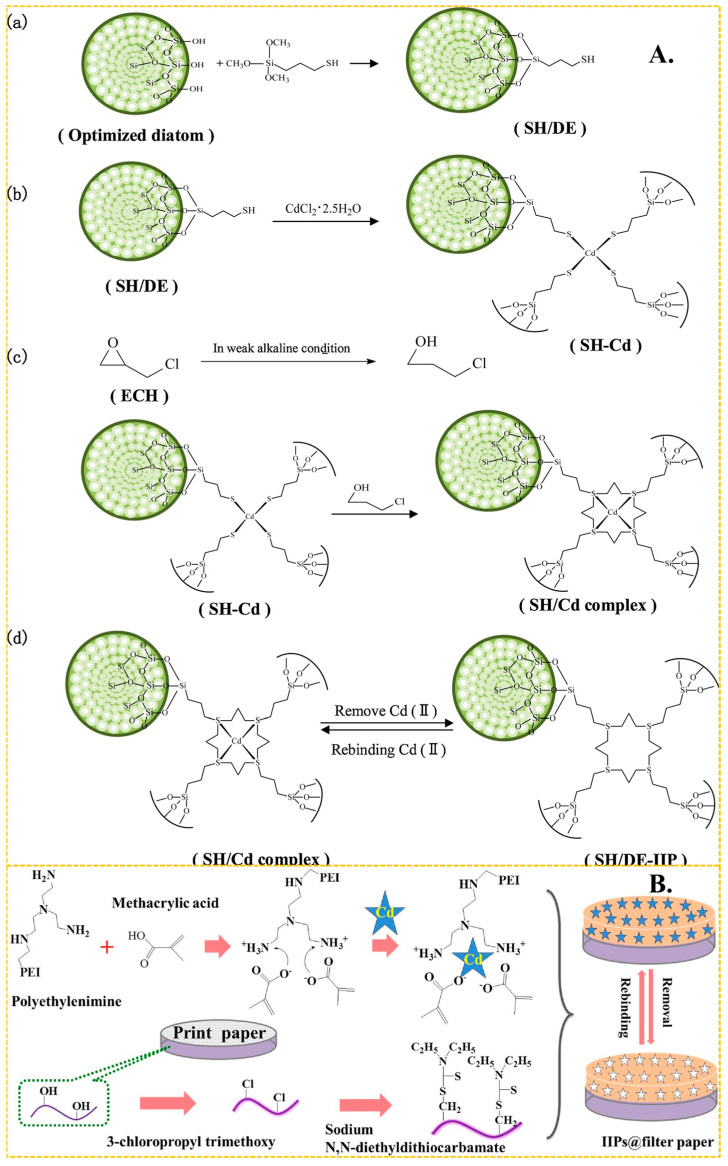
(**A**) Scheme for the synthesis of SH/DE-IIP, adapted with permission from Ref. [68] Copyright 2021 Elsevier. (**B**) Scheme for the synthesis of paper—based IIP, adapted with permission from Ref. [118]. Copyright 2017 Elsevier.

In recent years, (3-mercaptopropyl) trimethoxy silane has become one of the most popular monomers in the preparation of Cd(II)-imprinted polymers. It forms a Cd–SH bond with Cd(II) through the complexation of the lone pair electron of the S atom on the -SH group, and then produces mercaptan Cd-IIP through a series of steps including cross-linking and elution, to produce mercaptan Cd-IIP. As shown in Figure 4A, the grafting reaction can be reflected in Figure 4A(a). The crosslinker plays a role in immobilizing the Cd(II) and the MPS.The presence of Cd-S bonds can imply strong bonding interactions between Cd(II) and S atoms. The imprinting process is shown in Figure 4A(b), and the cross-linking reaction is shown in Figure 4A(c). It shows a good grafting environment on the diatom surface, forming stable imprinting sites, and the elution is mainly directed to Cd ions.The elution process of SH/DE-IIP can correspond to Figure 4A(d). Miao et al. [68] used thiol-modified diatom as carrier, (3-mercaptopropyl) trimethoxy silane as functional monomer, formed a S–Cd bond with the Cd(II), and took advantage of the surface active structure characteristics of diatom, and presents a three-dimensional cross-linked structure after cross-linking reaction, the maximum adsorption capacity of SH/DE-IIP was 4.8 mg/g, the removal rate of Cd(II) ions in aqueous solution was increased from 24.4% to 97%. Huang et al. [117] used the similar method synthetized Cd(II) IIP, the maximum adsorption capacity was up to 5.5025 mg/g. Anais Adauto et al. [65] utilized (3-mercaptopropyl) trimethoxy silane and 1-vinylimidazole as functional monomers to simultaneously bond Cd capacity of 4.73 mg/g, and the removal rate of Cd(II) from river water samples exceeded 90%.

According to the data in Table 4, it is evident that the adsorption efficiency of Cd(II) associated with functional monomers that contain amino, hydroxyl, and other groups is better than that of thiol groups. The maximum adsorption capacities of IIPs utilizing the aforementioned (3-mercaptopropyl) trimethoxy silane as functional monomers were mentioned to be relatively low, all below 10 mg/g. Cao et al. [119,120] utilized (3-mercaptopropyl) trimethoxy silane as the functional monomer, silica nanoparticles, and raspberry-like silica as the support. The carrier structure effectively increased the specific surface area of the materials and improved the diffusion kinetics of the polymer, resulting in the maximum adsorption capacities of these two IIPs achieving 22.6 mg/g and 36.62 mg/g, respectively. Lu et al. [121] employed sodium pyrrolidone carboxylate as the functional monomer to fabricate a magnetic mesoporous ion-imprinting adsorbent with a large specific surface area. Cd(II) was selectively adsorbed by the Cd–S bond, achieving a maximum adsorption capacity of 154.99 mg/g. Additionally, the presence of sodium pyrrolidone carboxylate imparted transparency to the materials, and upon contact with tetracycline, it could effectively degrade tetracycline with a degradation rate of 75.32%. This adsorbent could simultaneously degrade tetracycline while adsorbing Cd(II), offering a method to optimize the adsorption effect and multi-effect utilization of ion-imprinted adsorbent materials that rely on the Cd–S bond to chelate.

In addition to bonding with sulfhydryl groups, amino and hydroxyl groups also play a huge role in the preparation of Cd(II)-imprinted adsorption materials. These groups contribute to a high adsorption capacity and effective adsorption. Guo et al. [71] synthesized ion-imprinted polymers by using Fe_3_O_4_-g-C_3_N_4_ as a carrier and N-isopropylacrylamide as the functional monomer. The complexation of amino groups with Cd(II) resulted in a maximum adsorption capacity of 184 mg/g, and the adsorption capacity remained at 84% after 5 cycles. Zhu et al. [122] achieved a maximum adsorption capacity of 107 mg/g by bonding amino and hydroxyl groups with Cd(II) using the common functional monomer acrylamide. Later, Zhu et al. [123] synthesized functional monomers of Schiff bases using salicylaldehyde and ethylenediamine. These monomers bonded with Cd through amino and hydroxyl groups, and the final material, with ethylene glycol dimethacrylate as a cross-linker, exhibited a maximum adsorption capacity of 179.04 mg/g. Kai Huang et al. [118] used print paper as a carrier and MA and polyethylenimine as the functional monomer. As shown in Figure 4B, paper-based ion-imprinted polymers were obtained by reversible addition–fragmentation chain transfer polymerization, the maximum adsorption capacity was up to 155.2 mg/g, the imprinting factor (the ratio of the adsorption capacity of ion-imprinted polymers (MIP) for template ions to the adsorption capacity of non-ion-imprinted polymers (NIP) for template ions) was more than 3.0 and the limit of detection was 400 mg/mL.

**Table 4:** Composition and properties of Cd ion-imprinted polymer.

Carrier	Ligand	Functional Monomers	Group	Regeneration Frequencies	Maximum Adsorption Capacity (mg/g)	Ref.
CdS/Fe_3_O_4_	NA	sodium pyrrolidone carboxylate	-SH	10	154.99	[121]
NA	1-VI	MPS	-SH -NH	4	4.73	[65]
core-shell mesoporous silica nanoparticles	NA	MPS	-SH	5	22.6	[119]
activated diatomite	NA	MPS	-SH	5	5.5025	[117]
thiol-modified diatom	NA	MPS	-SH	5	4.8	[68]
Fe_3_O_4_@SiO_2_	NA	AECS	-NH_2_	6	26.1	[124]
Fe_3_O_4_@SiO_2_	PBTCA	NA	-COOH	6	29.82	[125]
NA	NA	TCCS	-SH	5	305	[126]
NA	NA	maleic anhydride; AN	C≡N C=O	10	20.46	[127]
Fe_3_O_4_@SiO_2_	NA	beer yeast	-NH	10	62.74	[128]
GO	NA	salecan	-COOH	5	412.5	[129]
NA	NA	AN; PA	-NH_2_ -OH C=N	NA	0.018	[130]
R_8_Si_8_O_12_	NA	1-VI; NMA	-C=O -OH C≡N	5	80.21	[131]
natural sand	NA	AM	-NH_2_ -C=O	8	33.84	[132]
NA	vim; MA		-NH -COOH		43	[133]
NA	MCO	MA	-COOH	10	62.9	[134]
NA	NA	β-cyclodextrin; AM	-NH_2_ -OH	5	107	[122]
Fe_3_O_4_	NA	salicylaldehyde Schiff base; MMA	-NH_2_ -OH	5	179.04	[123]

Abbreviations: PBTCA—2-Phosphonobutane-1,2,4-tricarboxylic acid; TCCS—thiosemicarbazide-chitosan derivative; AM—acrylamide; MA—methacrylic acid; MPS—(3-mercaptopropyl) trimethoxy silane; GO—graphene oxid; AN—acrylonitrile; AECS—aminoethyl chitosan; EDA—ethylenediamine; NMA—N-hydroxymethylacrylamide; vim/1-VI—1-vinylimidazole; NIAM—N-isopropylacrylamide; PEI—polyethylenimine; MMA—methyl methacrylate; PA—hydroxylamine hydrochloride; MCO—1-mercaptooctane.

### 3.4. Hg(II)-Imprinted Polymer

Hg(II) is highly neurotoxic, and its compounds can accumulate in organisms, causing chronic poisoning and health risks [135,136]. In the synthesis of Hg(II) ion-imprinted polymers, functional groups, like amino, hydroxyl, and thiol groups, are used for chelation.

For instance, a metal replaces an active hydrogen in an amine group, forming a chelate with a metal ion for increased stability. Esmali et al. utilized Hg(II) complexed with phenylphenanthroline as a template and acrylamide/acrylonitrile as functional monomers. The amine groups of the ligand and monomer coordinated with Hg(II) to prepare Hg(II) ion-imprinted polymers via free radical copolymerization [137]. Poly (ether sulfone)-based ion-imprinted films were prepared through phase conversion. When the concentration of Hg(II) was 4 mg/L, the maximum adsorption capacity was 432 mg/m^2^. Velempini et al. utilized cysteamine as a ligand to form a complex with Hg(II). In this process, the thiol group of cysteamine interacts with Hg(II) and connects the carboxymethyl cellulose polymer branch through an amide reaction with epichlorohydrin, resulting in the creation of Hg(II)-IIP [138]. When the concentration of Hg(II) is 400 mg/L, its maximum adsorption capacity is 80 mg/g. In the presence of Cu(II), Zn(II), Co(II), Pb(II), and Cd(II), it has high selectivity for Hg(II), and the recovery rates for Hg(II) containing wastewater, groundwater, and tap water are 86.78%, 91.88%, and 99.10%, respectively. Ruddy et al. created ion-imprinted polymers using 2-mercaptobenzoimidazole and 2-mercaptobenzothiazole as thiol ligands and acrylic acid as functional monomers. This IIP was designed to extract methylmercury from water samples [62]. When the starting concentrations were 0.46 mg/g and 0.06 mg/g, the highest adsorption capacities were 0.157 mg/g and 0.457 mg/g, respectively. This method has effectively been applied for removing methylmercury from samples of river and tap water. Rahman et al. created Hg(II)-IIP by utilizing Hg(II) as the template ion, [2-(methacryloxy) ethyl] trimethylcysteine as the ligand, and MAA as the functional monomer [139]. Simultaneously, the distinctive porous column resembling a pipette offered by the material addressed the challenges of making IIP suitable for commercial or industrial use. These ion-imprinted polymers, developed in recent years, involve Hg(II) binding with a single group and form coordination with Hg(II) using N or S ions in the molecular structure.

**Figure 5:**
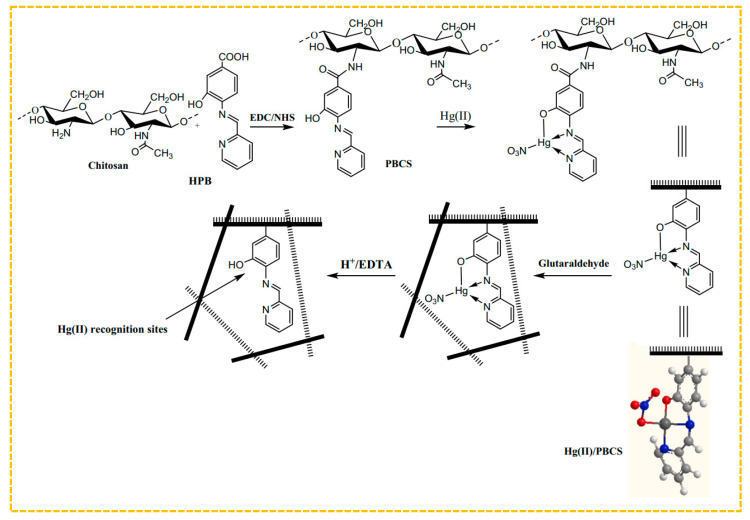
Scheme for the synthesis of Hg-S-IIPs, adapted with permission from Ref. [139]. Copyright 2022 Elsevier.

To improve the adsorption capability, combinations of amine, thiol, and hydroxyl groups can be utilized as ligands for metal ions. Hajri et al. created Schiff base ligands by using 4-amino-3-hydroxybenzoic acid and 2-pyridinecarboxylic aldehyde, and then connected the resulting modified chitosan polymer ligand with Hg(II) ions via amide bonds to form the polymer complex illustrated in Figure 5 [140]. The amine and hydroxyl groups were coordinated with Hg(II) and imprinted through glutaraldehyde cross-linking, eliminating the bound Hg(II) ions. Thus, the Hg(II)-imprinted adsorbent was ultimately obtained, with a maximum adsorption capacity of 315 mg/g at an initial concentration of 90 mg/L. Lins et al. created a sorbent for blotting Hg(II) by utilizing a bulk polymerization technique. They used a Hg(II)-disulfidehydrazone chelate formed from amine, sulfhydryl, and Hg(II) groups as a template, incorporated MAA as a monomer, and employed bulk polymerization to design an innovative online pre-enrichment system for selectively extracting and measuring Hg(II) in natural water samples [141].

From Table 5, it can be observed that due to the weaker anchoring of Hg(II) ions on the polymer chain compared to the use of S-based and N-based selective ligands, it is necessary to form a complex with the ligand before interacting with functional monomers containing vinyl groups. Due to the specific affinity of Hg(II) for sulfur, monomers containing thiol groups can form stable prepolymerization complexes with Hg(II). Reagents containing N-group functional groups are also prone to interact with Hg(II) through N-Hg N bonds. This provides us with a good direction on the choice of ligands for the preparation of Hg(II) ion-imprinted polymers in the future.

### 3.5. Pb(II)-Imprinted Polymers

As a specific pollutant in the aquatic environment, Pb(II) has garnered attention due to its high toxicity, resistance to biodegradation, and long-term adverse effects on human health and water systems [143,144].

**Figure 6:**
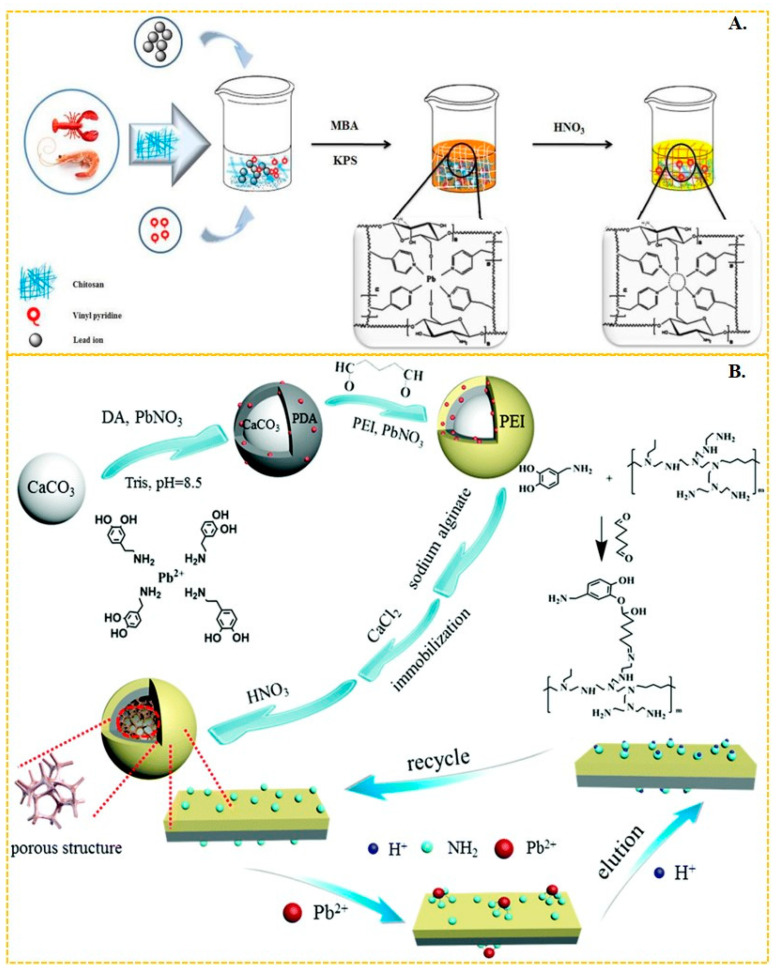
(**A**) Scheme for the synthesis of chitosan-based Pb-IIP, adapted with permission from Ref. [59]. Copyright 2020 Elsevier. (**B**) Scheme for the synthesis of Pb-IIP [147], adapted with permission from Ref. [147]. Copyright 2019 Royal Society of Chemistry.

Li et al. [58] synthesized Pb-IIP by using 4-vinylpyridine as the functional monomer and 2-(2-aminophenyl)benzimidazole as the ligand to form a magnetic layered graphene oxide composite. The N–Pb coordination bonding in this composite resulted in a maximum adsorbed amount of lead(II) of 58.82 mg/g. Similarly, Mohammad Landarani et al. [145] employed 4-vinylpyridine as the functional monomer and 2,6-diaminopyridine as the ligand to interact with lead(II), achieving a maximum adsorption capacity of 128 mg/g in their imprinted polymer. This polymer demonstrated excellent performance in the pre-enrichment of lead(II) in real water bodies. Javad Gatabi et al. [59] copolymerized chitosan-based Pb-IIP with 4-vinylpyridine as the functional monomer, resulting in a maximum adsorption of Pb(II) ions in aqueous solution up to 136 mg/g, as shown in Figure 6A. The 4-vinylpyridine molecule possesses an uninvolved sp^2^ hybridized orbital on the nitrogen atoms, which is occupied by a pair of lone electrons. This large electronegativity of the nitrogen atom enables it to form bonds with metal ions, making 4-vinylpyridine a commonly used functional monomer. Huang et al. [146] synthesized efficient surface Pb-IIPs based on sandwich graphene oxide composites. The amide bonds on the composites coordinated with Pb(II) through N and O. They utilized vinyl-modified graphene oxide with acrylamide as the functional monomer, resulting in a maximum adsorption capacity of 40.02 mg/g for these Pb-IIPs. Wang et al. [56] employed reverse suspension polymerization to prepare thermosensitive Pb-IIPs. Their material was based on multi-walled carbon nanotube composites with chitosan, hydroxyethyl methacrylate, and isopropyl acrylamide as monomers, which coordinated with Pb(II) through -NH, -NH_2_, and -COO. This Pb-IIPs exhibited a maximum adsorption capacity of Pb(II) up to 83.20 mg/g. Radhia Msaadi et al. [63] prepared ion-imprinted adsorbent/montmorillonite nanocomposites through photopolymerization in dimethyl sulfoxide using acrylamide and N, N′-methylene acrylamide as functional monomers. The adsorption capacity of these nanocomposites reached up to 301 mg/g through N–Pb bonding. Shen et al. [147] utilized polydopamine-polyethyleneimine-modified CaCO_3_ composites and sodium alginate as functional platforms to prepare a Pb-IIP (shown in Figure 6B) through chelation of -NH_2_ with Pb(II). This Pb-IIP displayed an adsorption capacity of up to 357.4 mg/g, achieving ultra-efficient and selective capture of Pb(II) from wastewater. Zhu et al. [148] also employed the ionic surface imprinting technique, synthesizing a two-dimensional montmorillonite-based surface ion-imprinted adsorbent with a maximum adsorption capacity of 201.84 mg/g. They achieved this by bonding the oxime group in salicyl hydroxamic acid to Pb(II) using montmorillonite as a carrier.

Combined with Table 6, it can be observed that in the preparation of Pb(II) ion-imprinted adsorbents, the nitrogen atoms in the functional groups are typically bonded with Pb(II), creating a specific structure in the IIP cavity that plays a selective role in the subsequent adsorption process. The commonly used functional monomers include 4-vinylpyridine, acrylamide, and vinylamide. With its double bond, 4-vinylpyridine allows for various addition and polymerization reactions. It can react with different functional monomers and cross-linkers to produce polymers with excellent performance. Its planar structure enables the formation of coordination bonds with Pb(II), resulting in high specificity and facilitating the highly selective adsorption and separation of target ions. Moreover, 4-vinylpyridine is relatively easy to synthesize.

### 3.6. Cr(VI)-Imprinted Polymers

Cr(VI) is one of the main toxic contaminants that cause environmental pollution and impact human health. Chromium metal exists in various forms in the environment, primarily as Cr (III) and Cr(VI) [157], while Cr (III) is a trace element necessary for the well-being of mammals, hexavalent chromium is known for its high carcinogenicity and toxicity, as it mutates genes and leads to diseases [158].

**Figure 7:**
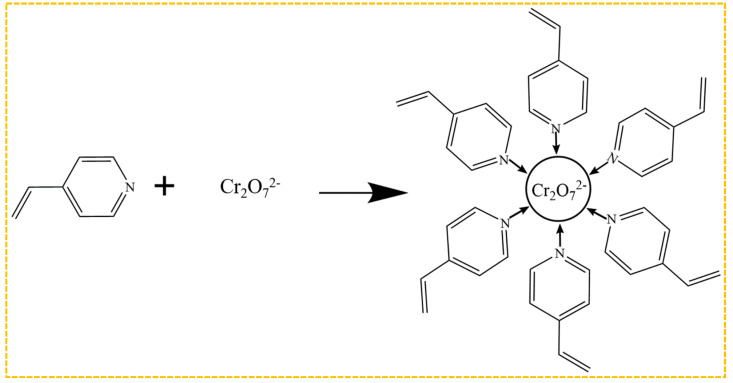
Mechanism of bonding of Cr_2_O_7_^2−^ in combination with 4-VP.

Amine polymers currently dominate as the functional monomers for the preparation of Cr(VI)-imprinted polymers, as illustrated in Figure 7. The imprinting sites are created by the bonding of amino groups to Cr_2_O_7_^2−^ after elution during the preparation process. Among these functional monomers, 4-vinylpyridine is the most commonly employed one, which interacts with Cr_2_O_7_^2−^ through the N atom on its tertiary amine. Zhou et al. [159] optimized the experimental conditions based on these findings, resulting in an enhanced adsorption capacity of 201.55 mg/g. Ren et al. [160] investigated the impact of eight functional monomers with varying acidity and alkalinity, including 4-vinylpyridine, acrylamide, methacrylic acid, and hydroxyethyl methacrylate, on the adsorption capacity of the materials. Among these, the Cr-IIP, prepared using 4-vinylpyridine as the functional monomer, exhibited the highest adsorption capacity, with a maximum adsorption amount of 338.73 mg/g. Additionally, it demonstrated good selectivity, reusability, and stability, which can be attributed to the electrostatic interactions between the Cr(VI) ions and the protonated N atom on the pyridine functional group of 4-vinylpyridine, thus enhancing both the adsorption capacity and rate.

In addition, to address the sensitivity of Cr_2_O_7_^2−^ to pH, it is worth considering the addition of corresponding ligands during the polymerization process. This would allow Cr(VI) to first form a stable complex, thereby enhancing its stability in a higher pH environment, before proceeding with the polymerization reaction. Samaneh Hassanpour et al. [161,162] prepared two types of magnetic Cr(VI) ion-imprinted adsorbents by modifying magnetic nanoparticles and magnetic multi-walled carbon nanotubes with 4-vinylpyridine as the ligand and hydroxyethyl methacrylate as the functional monomer. They also prepared two magnetic Cr(VI) ion-imprinted adsorbents using ethyl orthosilicate-modified magnetic nanoparticles and magnetic multi-walled carbon nanotubes, respectively. The maximum adsorption amounts for these adsorbents were 44.86 mg/g and 56.1 mg/g, which were relatively low. Liang et al. [163] used Fe_3_O_4_@SiO_2_ as a carrier and introduced graphene oxide to prevent Fe_3_O_4_@SiO_2_ aggregation and increase the specific surface area. They also used 4-vinylpyridine and hydroxyethyl methacrylate as ligands and functional monomers. The resulting ion-imprinted polymer exhibited a maximum adsorption capacity of up to 311.95 mg/g.

In addition to 4-vinylpyridine, another commonly employed functional monomer, 3-(2-aminoethyl)aminopropyltrimethoxysilane, is also a viable option. Huang et al. [164] fabricated Cr-IIP on graphene oxide-mesoporous silica nanosheets using the surface ion blotting technique, with propyltrimethoxysilane serving as the functional monomer for selective Cr(VI) adsorption through its amine group. The maximum adsorption amount achieved was 438.1 mg/g, and the material exhibited excellent reusability over five adsorption/desorption cycles. This approach holds great potential for treating Cr_2_O_7_^2−^ containing wastewater.

As demonstrated in Table 7, the vinyl pyridine monomer, which contains a C≡N group, has been widely employed as the functional monomer for Cr ion-imprinted polymers in recent years. This group ensures interaction with Cr, making it a popular choice. Another suitable option is propyltrimethoxysilane, which contains an -NH_2_ group. Carriers such as magnetic silica and carbon nanotubes offer a larger specific surface area for the ion-imprinted polymers, preventing agglomeration, increasing the number of recognition sites, and enhancing the adsorption performance of the material.

## 4. Metalloid Ion-Imprinted Polymer

Arsenic, antimony and other metal compounds are usually present in natural water as oxygenated anions or neutral molecules rather than metal cations. Metalloid elements are located in the metal-nonmetal border region of the periodic table, and their atomic radii are inherently larger than those of metal atoms in the same period. When these metalloid atoms lose electrons to form ions, the electron cloud is relatively loose due to their small number of valence electrons and their location on higher energy levels, which makes the radii of the metalloid ions relatively large. This results in a weak electrostatic effect between the metal-like compound and the functional monomer or ligand. Therefore, the fabrication of metal-like ion-imprinted adsorbents is challenging and reports in this regard are limited [180].

### 4.1. As(III)-Imprinted Polymer

Due to its contamination of groundwater sources, arsenic poses a significant health risk and is a major global concern for countries addressing water-related issues. In natural water sources, arsenic exists in two main forms: arsenite (AsO_3_^3−^) and arsenate (AsO_4_^3−^), also known as As(III) and As(V) [181]. Arsenic in the form of As(III) is more harmful and has carcinogenic properties compared to As(V) [182,183]. Prolonged exposure to water contaminated with As(III) can result in kidney and nervous system disorders, while even consuming small quantities of As(III) can be fatal [184,185].

As(III) binds to a specific type of functional group through chelation and then creates a polymer by linking with a cross-linking agent. Chi et al. employed As(III) as the template ion and methacrylic acid as the functional monomer. The methacrylic acid coordinated with As(III) via its carboxyl group and was subsequently cross-linked to Fe_3_O_4_/graphene oxide using ethylene glycol dimethacrylate to create a novel surface ion-imprinting adsorbent [186]. When the initial concentration of As(III) was about 80 mg/L, the maximum fitted adsorption capacity of the material was 49.42 mg/g. Yin et al. created mesoporous imprinted polymers by utilizing As(V) as the template, diethylenetriamine, and 3-(2-aminoethyl) aminopropyltrimethoxysilane as ligands and functional monomers. This was achieved through the coordination of amino groups and template ions [187]. The polymers’ ability to adsorb As(V) reached its maximum at a concentration of 2000 mg/L, with an adsorption capacity of 78.74 mg/g. When applied to real water samples, the polymers showed recovery rates ranging from 81.8% to 95.4%. Even after six consecutive uses, the polymers maintained an adsorption capacity of 93.0%.

**Figure 8:**
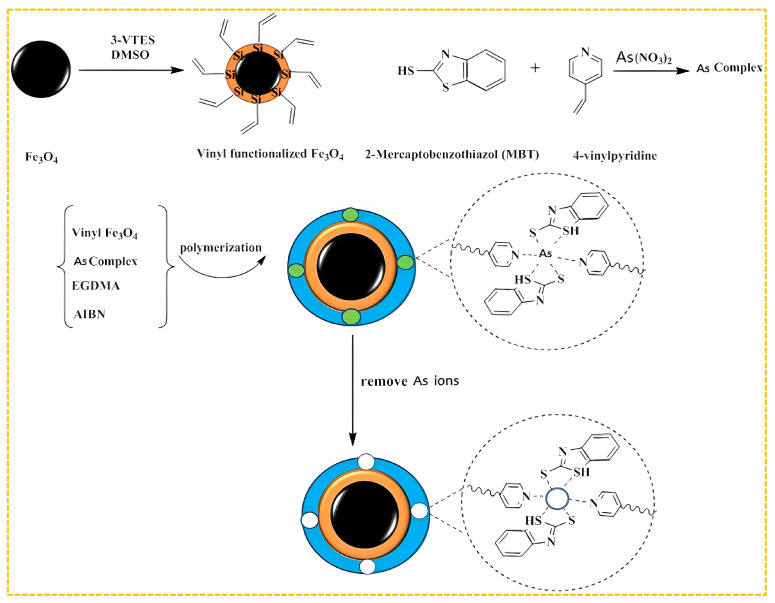
Scheme for the synthesis of IIP@SiO_2_@Fe_3_O_4_, adapted with permission from Ref. [189]. Copyright 2020 Elsevier.

In order to increase the number of different types of imprinted holes, two ligands or monomers that chelate with arsenic ions can be combined to form a double-imprinted ion polymer. Samah et al. synthesized As(III)-IIP for arsenic removal from water using As(III) as a template and allyl thiourea as the functional monomer, with amine and thiol groups coordinated with template ions [188]. For an initial concentration of 25 mg/L As(III), the adsorption capacity was 7.255 mg/g. Sadani et al. used As(V) as the template ion, 2-mercaptobenzothiazole as the ligand, 4-vinylpyridine as the functional monomer, methacrylate as the cross-linking agent, azodiisobutyronitrile as the initiator, and magnetic silica nanoparticles as the carrier [189]. Amine and thiol groups were coordinated with the template ion to prepare IIP@SiO_2_@Fe_3_O_4_ granule. When the concentration of As(V) is 6.28 mg/L, the adsorption amount can reach 104.7 mg/g, with an adsorption efficiency of 97.46%. IIP is supported by SiO_2_ nanoparticles to generate a larger surface area and more adsorption sites. The preparation process is shown in Figure 8. Jagirani et al. used As(III) as templates, 4-vinylpyridine, and 2-hydroxyethyl methacrylate as ligands and functional monomers [57]. Amine and hydroxyl groups were used in coordination with template ions to create an As(III)-IIP through a co-precipitation technique. At an initial As(III) concentration of 5 mg/L, the highest adsorption capacity of the IIP was found to be 106.3 mg/g. The effective adsorption rate for As(III) in water samples was approximately 99%.

As shown in Table 8, for As(III)- or As(V)-imprinted polymers, the O atoms in commonly used template ions arsenite and arsenate ions can interact well with reagents containing hydroxyl groups, as well as effectively with N groups in vinyl pyridine monomers. While the oxygen anion configurations of As(III) and As(V) can effectively bond with standard bifunctional ethylene monomers, incorporating suitable ligands can enhance the material’s adsorption capabilities and selectivity to some degree. From the table, it can be seen which using (3-mercaptopropyl) trimethoxy silane as the functional monomer and magnetic graphene oxide as the carrier can significantly increase the adsorption capacity of the synthesized ion-imprinted polymer. Graphene oxide is costly, whereas (3-mercaptopropyl) trimethoxy silane is less expensive but lacks environmental friendliness. Therefore, the emphasis can be placed on utilizing cost-effective and eco-friendly materials like magnetic silica carriers and 4-vinylpyridine monomers to advance the industrial use of ion-imprinted materials.

### 4.2. Sb(III)-Imprinted Polymer

Antimony is a toxic metalloid substance that exists at extreme trace levels in the environment [192]. Antimony compounds in the III oxidation state are more harmful than those in the V oxidation state, with Sb(III) being ten times more toxic than Sb(V). This is significant because antimony trioxide is commonly used as a catalyst for polyethylene terephthalate (PET) in plastic bottles [193], and since PET is widely used for packaging beverages and water, antimony is often found in bottled water and beverages. Antimony, like arsenic, is a thiol-based poison that can attach to the thiol groups of specific enzymes in the body, disrupt tissue metabolism, and harm the heart, liver, kidneys, and nervous system [194].

**Figure 9:**
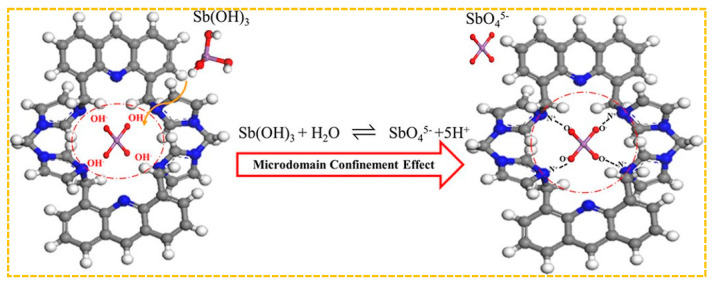
Adsorption mechanism of Sb—CFM—II. Adapted with permission from Ref. [180] Copyright 2018 American Chemical Society.

Shakerian et al. created a specialized adsorbent by using antimony (III) as a template, pyrrolidine dithiocarbamate as a ligand, and styrene as a monomer. This was achieved by coordinating C–S bonds with antimony (III) [195]. When the initial concentration of Sb(III) was 20 mg/L, the adsorption capacity was 6.7 mg/g, which can be reused for at least ten cycles. Jakavula et al. prepared Sb(III)-IIP using magnetic mesoporous silica carbon fiber(Fe_3_O_4_@CNFs@SiO_2_) nanocomposites as support substrates and surface imprinting technology based on the above synthesis method [196]. The rationale behind the selection of carbon nanofibers lies in their extensive surface area, robust interactions with diverse substances, and strong affinity for metals. Moreover, the integration of magnetic nanomaterials within a carbon nanofiber framework addresses the limitations associated with inadequate separability and reproducibility when utilizing carbon nanofibers as a support for ion-imprinted polymers, thereby enhancing separation efficiency and recyclability. Furthermore, the amalgamation of mesoporous silica with magnetic carbon fibers yields superior IIP carriers characterized by a substantial surface area and customizable pore size. For instance, at an initial Sb(III) concentration of 8 mg/L, the saturated adsorption capacity is measured at 47.8 mg/g. This underscores that the combination of imprinted polymers with carriers can augment porosity and specific surface area, furnish additional metal ion binding sites, and consequently elevate adsorption capacity.The absence of practical functional monomers presents difficulty in creating metalloid imprinting materials. Fang et al. developed a new positively charged cyclic functional monomer, tetrabromobiphenyl-4,5-di(methylenebisimidazole) acridine, to be used in the production of Sb-CFM-IIP [180]. The adsorption mechanism is shown in Figure 9. The chemisorption process of Sb(III) on CFMIIP may be: (a) Sb(OH)_3_ enters the nanoscale imprinted cavity of CFM-IIP; (b) Sb(OH)_3_ is hydrolyzed to SbO_4_^5−^ when it meets with hydroxyl radicals released from CFM-IIP under specific microstructural domain restriction conditions (these hydroxyl radicals are produced by replacing the previous Sb(III) template after base elution), (c) The negatively charged O of SbO_4_ ^5−^ is rapidly captured by the positively charged N of CFM-IIP. The nanoscale imprinting cavity of CFM-IIP generates a domain confinement effect, promoting the hydrolysis of Sb(OH)_3_ into SbO^5−^. Due to the strong electrostatic attraction and size matching of CFM-IIP and SbO^5−^, SbO^5−^ is then isolated in the imprinting cavity of CFM-IIP. When the initial concentration of Sb(III) in the solution is 350 mg/L, the maximum adsorption capacity of CFM-IIP for Sb(III) is 79.1 mg/g, while the adsorption capacity of non-cyclic functional monomer imprinted polymer(NCFM-IIP) is only 30.9 mg/g. Therefore, CFM has better performance compared to NCFM-based adsorbents.

According to the data presented in Table 9, the molecularly imprinted polymer (MIP), derived from styrene as the functional monomer, exhibits lower selectivity and adsorption capacity towards Sb(III) compared to imidazole. In contrast, imidazole demonstrates superior imprinting efficiency and arsenide binding capabilities. The IIP prepared from tetrabromobiphenyl-4,5-di (methylene bis imidazole) acridine as a functional monomer has a high selectivity and adsorption capacity for Sb(III), mainly due to the large ring effect of CFM, which has a strong affinity for oxygen anions through size matching, and the electrostatic double effect generated by its positively charged imidazole ring and appropriate ring size, which also provides ideas for the preparation of oxygen anionic imprinting materials.

## 5. Dual/Multi-Ion-Imprinted Polymers

In practice, metal pollution is usually a composite pollution of several metal ions [198,199]. In the analysis of trace metals, the challenges of accurately detecting target ions are compounded by their extremely low concentrations, potential cross-reactions with multiple ions, and interference from matrix components [200]. Hence, there is a need to create specialized adsorbents that possess multiple selective recognition capabilities for the purpose of extracting analytes, purifying samples, and concentrating them prior to analysis.

**Figure 10:**
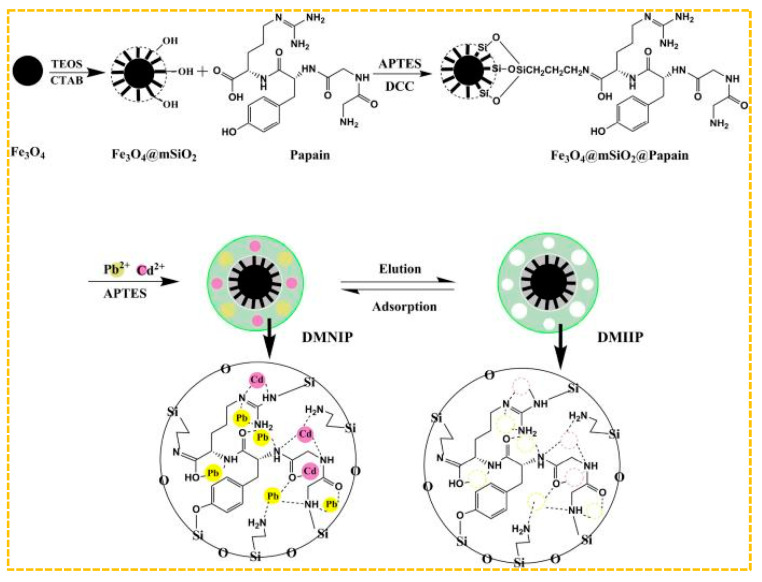
Scheme for the synthesis of paper-based DMIIP, adapted with permission from Ref. [202]. Copyright 2020 Elsevier.

Mehdi et al. adopted the method of combining hard and soft templates, using N-[(3-Trimethoxysilyl) propyl] ethylendiamine triacetic acid trisodium salt as Cd(II)’s functional monomer, methacrylic acid as Pb(II)’s functional monomer, and hierarchical silica-based-imprinted mesoporous polymers [201]. The polymers demonstrated high recovery rates for Cd(II) and Pb(II) at 99.48% and 98.03%, respectively. Additionally, the imprinting factors for Cd(II) and Pb(II) were found to be 11.90 and 12.69, respectively. Ultimately, the examination of Cd(II) and Pb(II) in river water and fish samples was conducted through the utilization of polymers. Furthermore, Prasad et al. developed a dual ion-imprinted polymer incorporated within a sol-gel matrix, with acrylic acid serving as the functional monomer [200]. It can be used to modify solid sensors to quantitatively detect ultra-trace amounts of Cd(II) and Cu(II) simultaneously, avoiding the bias caused by the metal–metal interaction between Cd(II) and Cu(II) in real samples. As shown in Figure 10, Xie et al. employed magnetic mesoporous Fe_3_O_4_@mSiO_2_ as a carrier and substituted organic amines with papain as functional monomers. They utilized a surface imprinting technique in conjunction with a sol-gel process to fabricate an ion-imprinted polymer (DMIIP) specific for Cd(II) and Pb(II), Cd^2+^ in purple, Pb^2+^ in yellow, and dual template ion imprinting removes both ions simultaneously by adsorption. [202]. Papain exhibits metal binding capabilities as a result of possessing numerous active binding sites, including -NH_2_, -OH, and =CO, thereby contributing to a reduction in the expenses associated with industrial manufacturing processes. The highest adsorption capacity of DMIIP for Cd(II) was found to be 41.69 mg/g, while for Pb(II) it was 76.39 mg/g. This facilitated the efficient separation and concentration of Cd(II) and Pb(II) in environmental and food samples. Hashami et al. used silica-coated Fe_3_O_4_ nanoparticles as a carrier, benzyl bis(thiosemicarbazone) ligand as the ligand, and obtained a dual-template-imprinted polymer Fe_3_O_4_@SBA-15-NH_2_-IIP [203]. Hossein et al. used 1,10-phenanthroline as the functional monomer, a magnetic graphene oxide as the carrier, and synthesized a dual-template-imprinted polymer for Cd(II)/Ni(II); this polymer is used for the pre-enrichment and determination of Cd(II) and Ni(II) in water samples [204].

Currently, there is a scarcity of research on multi-element analysis employing imprinted polymers. The existing studies predominantly focus on dual-ion imprinting, with limited exploration of adsorbents involving more than two types of ion imprinting [205,206,207,208,209]. Fu et al. first used 3-aminopropyltriethoxysilane as the functional monomer, tetraethoxysilicane as the cross-linker, dithizone as the chelator, and Hg(II), Ni(II), Cu(II), and Cd(II) as templates to insert into multi-ion-imprinting adsorbent [210]. It has been used as a solid phase extraction adsorbent for the preconcentration of trace Hg(II), Ni(II), Cu(II) and Cd(II) in seawater samples with a high detectability up to 6.0–22.5 ng/L and a recovery rate of 94.7~110.2%. Jakavula et al. employed the sol-gel technique for the production of a multi-ion-imprinting adsorbent. This involved utilizing (3-aminopropyl) triethoxysilane as the functional monomer, tetraethyl orthosilicate as the cross-linker, and ammonium pyrrolidine dithiocarbamate as the chelator [211]. It is used as an adsorbent for simultaneous extraction and enrichment of potentially toxic metal ions Sb(III), Te(IV), Pb(II), and Cd(II) in the matrix, combined with inductively coupled plasma emission spectroscopy. Under optimum conditions, the enhancement factors limit of detection and limit of quantification were 37.7–51.1, 0.04–100 µg/L, 0.011–0.28 µg/L, and 0.037–0.93 µg/L, respectively.

Currently, there is limited literature available on multi-ion-imprinted polymers, with the predominant use of functional monomers being acrylic and oxy-silane materials, which are considered overly simplistic. Nevertheless, there will be a competitive environment among various multi-template ions, with factors such as ion radius and electron configuration influencing the adsorption efficiency of the eventual IIP, thereby exerting a significant influence. Hence, the careful choice of functional monomers and ligands, the proportion of target ions, and the sequence of their introduction are crucial factors to consider.

## 6. Summary and Outlook

In recent decades, with the in-depth research on water treatment technology, the exploration of multifunctional, efficient, economical, and sustainable methods for heavy metal ion wastewater purification has increased. Among these methods, ion-imprinting technology to specifically recognize template ions for an efficient and synergistic recovery of the ions has been a hot topic. In this work, the research progress on the adsorptive removal properties of several heavy metal ions and metal-like ions by typical ion-imprinted methods has been summarized. The mechanisms, as well as applications of these ion-imprinted polymers, were discussed in detail.

The most commonly used functional monomers in the preparation of ion-imprinted adsorbents include 4-vinylpyridine, acrylamide, methacrylic acid, acrylic acid, chitosan, and (3-mercaptopropyl)trimethoxysilane. These functional monomers are coordinated to metals via N, O, and S atoms in the amino, hydroxyl, and sulfhydryl groups. However, the final ion-imprinted adsorption also depends on the type and amount of solvents, cross-linkers, carriers, and other compositions. Considering the current rate of development and application of ion blotting technology, future research prospects are as follows:

(1) Multi-template imprinting allows for the simultaneous removal of more types of contaminants than single-template imprinted polymers. From the perspective of wastewater resource utilization, a pollutant treatment process with high selectivity is the most promising strategy.
(2) Currently, low-cost environmentally friendly materials, such as chitosan and corn stover, are gradually being used in the field of water treatment. Based on this, we should explore the design of environmentally friendly and green functional monomers, as well as the introduction of responsive elements such as photosensitivity and thermal sensitivity, to prepare stimulus-responsive “smart” imprinted materials.
(3) The water samples used in most of the adsorption experiments are ideal solutions prepared in the laboratory. Interactions between concurrent pollutants in heavy metal industrial wastewater may affect the adsorption of ion-imprinted polymers through synergistic effects. Therefore, systematic studies are valuable to provide the necessary research data for their industrial applications.

## Figures and Tables

**Table 1:** Comparison of heavy metal ion adsorption performance of different materials.

Material Type	Pollutant	Maximum Adsorption Capacity (mg/g)	Adsorption Efficiency (%)	Ref.
Magnetism COF	Pb(II)	411.80	95.64	[36]
Biomass Charcoal Composites MgO@ZnO@BC	Cu(II)	50.63	93.25	[37]
Zr-MOFs	Cu(II)	9.78	97.80	[38]
PDA/MgAl-LDH	Cr(VI)	87	93.37	[39]
PPM-PVAm	Cr(VI)	208.3	90.6	[40]
shrub biological agent	Pb(II)	63.77	92	[41]
Y-type zeolite	Cd(II)	53.58	80	[42]
Iron-modified zeolite nanocellulose membrane	Ni(II)	7.46	85	[43]
Bacterial cellulose membrane	Ni(II)	28.18	92.95	[44]
Magnetic microcrystalline cellulose/MoS_2_/Fe_3_O_4_	Hg(II)	469.48	95.64	[45]
UiO-66-NH_2_	Pb(II)	200.17	92.31	[46]
chitosan	Ni(II)	87.45	94	[47]

**Table 3:** Composition and properties of nickel ion-imprinted polymers.

Carrier	Ligand	Functional Monomers	Group	Regeneration Frequencies	Maximum Adsorption Capacity (mg/g)	Ref.
CoFe_2_O_4_@MPS	NA	AM; SA	-CONH_2_	4	41.95	[101]
silica-coated magnetic Fe_3_O_4_@SiO_2_	4-VP	MA	C≡N -CO-OH	10	158.73	[66]
Fe_3_O_4_@GO	NA	AMPS	-N-C=O	10	35.31	[102]
Fe_3_O_4_@SiO_2_	NA	AMPS	-N-C=O	6	44.64	[103]
NA	NA	NDTEA	-NH	4	5387	[104]
NA	NA	N-(2-hydroxyphenyl) acrylamide	-NH -OH	6	38	[105]
inorganic mesoporous silica	BIDA	NA	C≡N -COOH	7	167.55	[106]
NA	4-VP	MA	C≡N -COOH	10	125	[55]
Fe_3_O_4_	NA	CTS	-NH_2_ -OH	15	18.5	[107]
NA	NA	CTS	-NH_2_ -OH	5	20	[97]
magnetic carbon nanotubes	NA	AA; CTS	-NH_2_ -COOH	5	19.86	[67]
OS	NA	CMC	-NH_2_ -COOH	6	69.1	[108]
NA	NA	CTS	-NH_2_	5	69.93	[109]
silica gel	NA	AAAPTS	-NH_2_ -NH	10	14.93	[110]
CoFe_2_O_4_/Bentonite	NA	VETOS	-OH	5	16.51	[111]
CoFe_2_O_4_/Bentonite	NA	PVA	-OH	5	11.77	[112]
SG-PMS	NA	AMPS	-SOOOH	6	20.3	[100]

Abbreviations: AM—acrylamide; MA—methacrylic acid; BIDA—2,2′-Biquinoline-4,4′-dicarboxylic acid; 4-VP—4-vinylpyridine; PVA—polyvinyl alcohol; VETOS—triethoxysilane; AMPS—2-Acryloyl-2-methylpropionic acid; NDTEA—N-(1-(2,4-difluorophenyl)-2-(1H-1,2,4-triazol-1-yl)ethyl)acrylamide; CTS—chitosan; OS—oyster shell; CMC—carboxymethyl chitosan; AAAPTS—3-[2-(2-aminoethylamino) ethylamino]propyl-trimethoxysilane; AA—acrylic acid; HEMA—hydroxyethyl methacrylate; SG-PMA—N-propylmaleamic acid-functionalized silica gel; SA—sodium acrylate; DMO—Diacetyl monoxime.

**Table 5:** Composition and properties of mercury ion-imprinted polymers.

Ligand	Functional Monomers	Group	Regeneration Frequencies	Maximum Adsorption Capacity (mg/g)	Ref.
Phenylphenanthroline	AM	-NH -NH_2_ C≡N	6	21.6	[137]
Cysteamine	NA	-SH -NH_2_	5	80.0	[138]
NA	4-VP	C≡N	NA	31	[142]
2-mercaptobenzothiazole	AA	-SH	NA	0.457	[62]
Schiff base	NA	R-N S-OH	5	315	[140]

Abbreviations: AA—acrylic acid; AM—acrylamide; 4-VP-4-vinylpyridine.

**Table 6:** Composition and properties of lead ion-imprinted polymers.

Carrier	Ligand	Functional Monomers	Group	Regeneration Frequencies	Maximum Adsorption Capacity (mg/g)	Ref.
NA	4-VP	2,6-DAPy	C≡N -NH_2_	5	128	[145]
NA	APBI	4-VP	C≡N -NH_2_	5	58.82	[58]
CS	NA	4-VP	C≡N -OH	5	136	[59]
Fe_3_O_4_	NA	4-VP	C≡N	5	123.3	[149]
CaCO_3_ composite materials	NA	4-VP	C≡N	5	357.4	[147]
Montmorillonite	NA	4-VP	-C=N -OH	6	201.84	[148]
MWCNTs	NA	4-VP	C=O	6	18.09	[150]
NA	NA	CTS; serratia marcescens	-NH_2_ -CO	5	116.279	[151]
magnetic multi-walled carbon nanotubes	DTZ	MAPTMS; AM	-NH -C=S	6	80.81	[152]
NA	NA	CTS; NSB	-SH -NH_2_	5	300	[153]
Fe_3_O_4_	NA	ITA	C-O	5	26.4	[154]
Diatomaceous earth	NA	MPTES	-SH	6	79.38	[155]
magnetic starch	1,10-phenanthroline	NA	C≡N	5	120	[156]
NA	Dz	AM	-NH-S-	NA	301	[63]

Abbreviations: 4-VP—4-vinylpyridine; AM—acrylamide; DTZ—Dithizone; MAPTMS—methacryloxypropyl trimethoxysilane; CTS—chitosan; NSB—3-Nitro-4-sulfanylbenzoic acid; ITA—itaconic acid; MPTES—3-mercaptopropyltriethoxysilane; Dz—Dithihydrazone; APBI—2-(2-Aminophenyl)-1H-benzizole; 2,6-DAPy—2,6-Diaminopyridine.

**Table 7:** Composition and properties of Cr ion-imprinted polymers.

Carrier	Ligand	Functional Monomers	Group	Regeneration Frequencies	Maximum Adsorption Capacity (mg/g)	Ref.
NA	NA	4-VP	C≡N	5	338.73	[160]
NA	phen	ST; 4-VP	C≡N	NA	0.41	[165]
NA	phen	ST	C≡N	NA	1.18	[165]
APTES	NA	4-VP	C≡N	5	56.46	[166]
Magnetic nanoparticles	4-VP	HEMA	C≡N	5	44.86	[161]
MMWCNTS	4-VP	HEMA	C≡N	5	56.1	[162]
CAB	NA	PEI	-NH -NH_2_	6	679.13	[167]
NA	2,2-(azanediylbis (ethane-2,1-diyl)) bis (isoindoline-1,3-dione)	MA	-CONH-	6	74.65	[168]
PP	NA	GAM	-NH -NH_2_	NA	103	[169]
Bisphenol A epoxy resins	NA	MA; EDA	-NH_2_ -CONH- -OH	5	263.15	[170]
MBA-15	NA	MA; 4-VP	-CONH C≡N	5	96.32	[171]
nylon-6	NA	4-VP	C≡N	NA	1.799	[172]
NA	NA	4-VP	C≡N	10	3.28	[173]
CTS	HRAB	NA	C≡N -OH	5	293	[174]
Polypropylene fibers	NA	AM; GMA	C=O -NH_2_	6	43.2	[175]
Nylon membranes	NA	ADPD	C=O	5	30.35	[176]
NA	NA	PVA; SA	-COOH -OH	3	1.75	[177]
PP	NA	AA; TETA	-NH_2_	10	167	[178]
PP	NA	ECH; DMAEMA	-NH C=O	5	156.5	[179]

Abbreviations: 4—VP-4-vinylpyridine; HEMA—hydroxyethyl methacrylate; CAB—aluminum-gelled carboxymethyl cellulose microspheres; PEI—polyethyleneimine; AA—acrylic acid; TETA—triethylenetetramine; SA—sodium alginate; PVA—poly(vinyl alcohol); AM—acrylamide; MA—methacrylic acid; GMA—glycidyl methacrylate; PP—polypropylene fibers; ECH—epichlorohydrin; DMAEMA—2-(dimethylamino)ethyl methacrylate; SBA-15—mesoporous silicon; CTS—chitosan; HRAB—azo dye; nylon-6—nylon filter membrane; ADPD—namely 2-allyl-1,3-diphenylpropane-1,3-dione; APTES—(3-aminopropyl) triethoxysilane; phen—1,10-phenanthroline complex; ST—styrene.

**Table 8:** Composition and properties of arsenic ion-imprinted polymers.

Carrier	Ligand	Functional Monomers	Group	Regeneration Frequencies	Maximum Adsorption Capacity (mg/g)	Ref.
Magnetic graphene oxide	NA	MA	-COOH	5	49.42	[186]
NA	NA	allyl thiourea	C=S -NH -NH_2_	NA	7.255	[188]
NA	Diethylenetriamine	[3-(2-Aminoethyl)aminopropyl]trimethoxysilane	-NH	6	78.74	[187]
modified hydrophobic Fe_3_O_4_ nanoparticles	2-acetyl benzofuran thiosemicarbazone	MA	C=S -NH_2_	5	37.04	[190]
Vinyl modified magnetic silica	2-mercaptobenzothiazole	4-VP	C≡N C=S -SH	4	104.7	[191]
NA	4-VP	2-HEMA	C≡N -OH	10	106.3	[57]

Abbreviations: MA-methacrylic acid; 4-VP-4-vinylpyridine; HEMA—hydroxyethyl methacrylate.

**Table 9:** Composition and properties of antimony ion-imprinted polymers.

Carrier	Ligand	Functional Monomers	Group	Regeneration Frequencies	Maximum Adsorption Capacity (mg/g)	Ref.
NA	1-Pyrrolidinecarbodithioic acid	Styrene	C-S	10	6.7	[195]
Magnetic mesoporous silica carbon fiber	1-Pyrrolidinecarbodithioic acid	Styrene	C-S	7	47.8	[196]
SA	NA	hyperbranched polyamide	-CONH_2_ -NH_2_	8	35.57	[197]
NA	NA	Tetrabromobiphenyl-4,5-di (methylenebisimidazole) acridine	C≡N	NA	79.1	[180]

Abbreviations: SA—Sodium alginate; MA—methacrylic acid.
